# Bis(μ-pyridinium-2-carboxyl­ato-κ^2^ *O*:*O*′)bis­[triaqua­(sulfato-κ*O*)manganese(II)]

**DOI:** 10.1107/S1600536811054596

**Published:** 2011-12-23

**Authors:** Hossein Ali Rasekh, Bohlul Bahrami

**Affiliations:** a Department of Chemistry, Islamic Azad University, Firoozabad Branch, Firoozabad, Fars, Iran

## Abstract

The asymmetric unit of the title compound, [Mn_2_(SO_4_)_2_(C_6_H_5_NO_2_)_2_(H_2_O)_6_], comprises half of a centrosymmetric dimer. The Mn^II^ atom is coordinated by two O atoms of the monodentate carboxyl­ate ligand, an O atom of the sulfate anion in axial position and three water mol­ecules in a distorted octa­hedral geometry. In the crystal, mol­ecules are connected through N—H⋯O and O—H⋯O hydrogen bonds, forming a three-dimensional network. The crystal structure is further stabilized by inter­molecular π–π inter­actions [centroid–centroid distance = 3.842 (2) Å].

## Related literature

For standard bond lengths, see: Allen *et al.* (1987 ▶). For background to the applications of Mn^II^ complexes, see: Lee *et al.* (2004 ▶); Mautner *et al.* (1997 ▶).
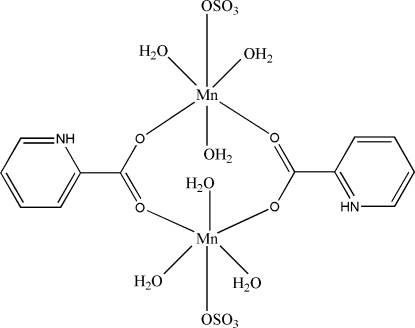


## Experimental

### 

#### Crystal data

[Mn_2_(SO_4_)_2_(C_6_H_5_NO_2_)_2_(H_2_O)_6_]
*M* *_r_* = 656.32
Orthorhombic, 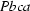

*a* = 16.886 (3) Å
*b* = 7.6022 (15) Å
*c* = 18.070 (4) Å
*V* = 2319.6 (8) Å^3^
*Z* = 4
Mo *K*α radiation
μ = 1.36 mm^−1^
*T* = 291 K
0.28 × 0.22 × 0.18 mm

#### Data collection

Bruker SMART APEXII CCD area-detector diffractometer
Absorption correction: multi-scan (*SADABS*; Bruker, 2005 ▶) *T*_min_ = 0.702, *T*_max_ = 0.792
5787 measured reflections
1945 independent reflections
1386 reflections with *I* > 2σ(*I*)
*R*_int_ = 0.033

#### Refinement

*R*[*F* ^2^ > 2σ(*F* ^2^)] = 0.028
*wR*(*F* ^2^) = 0.075
*S* = 1.26
1944 reflections
181 parameters
9 restraints
H atoms treated by a mixture of independent and constrained refinement
Δρ_max_ = 0.28 e Å^−3^
Δρ_min_ = −0.24 e Å^−3^

### 

Data collection: *APEX2* (Bruker, 2005 ▶); cell refinement: *SAINT* (Bruker, 2005 ▶); data reduction: *SAINT*; program(s) used to solve structure: *SHELXTL* (Sheldrick, 2008 ▶); program(s) used to refine structure: *SHELXTL*; molecular graphics: *SHELXTL*; software used to prepare material for publication: *SHELXTL* and *PLATON* (Spek, 2009 ▶).

## Supplementary Material

Crystal structure: contains datablock(s) global, I. DOI: 10.1107/S1600536811054596/bq2328sup1.cif

Structure factors: contains datablock(s) I. DOI: 10.1107/S1600536811054596/bq2328Isup2.hkl

Additional supplementary materials:  crystallographic information; 3D view; checkCIF report

## Figures and Tables

**Table 1:** Hydrogen-bond geometry (Å, °)

*D*—H⋯*A*	*D*—H	H⋯*A*	*D*⋯*A*	*D*—H⋯*A*
N1—H1⋯O5^i^	0.86	1.97	2.799 (3)	161
O1*W*—H11⋯O5^ii^	0.81 (2)	2.06 (2)	2.856 (3)	168 (3)
O1*W*—H12⋯O5^i^	0.84 (2)	1.91 (2)	2.735 (3)	173 (3)
O2*W*—H21⋯O4^ii^	0.82 (2)	1.90 (2)	2.704 (3)	167 (4)
O2*W*—H22⋯O4^iii^	0.83 (2)	1.97 (2)	2.758 (3)	158 (3)
O3*W*—H31⋯O6^ii^	0.82 (2)	1.92 (2)	2.736 (3)	177 (4)
O3*W*—H32⋯O6^iv^	0.83 (2)	1.84 (2)	2.660 (3)	172 (3)

Symmetry codes: (i) 
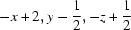
; (ii) 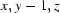; (iii) 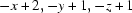; (iv) 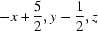.

**Fig. 1.**
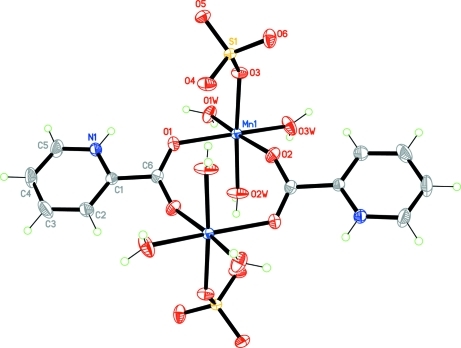
The molecular structure of the title compound, with 40% probability displacement ellipsoids. Symmetry code for the unlabeled atoms: -x + 2, -y + 1, -z + 1

**Fig. 2.**
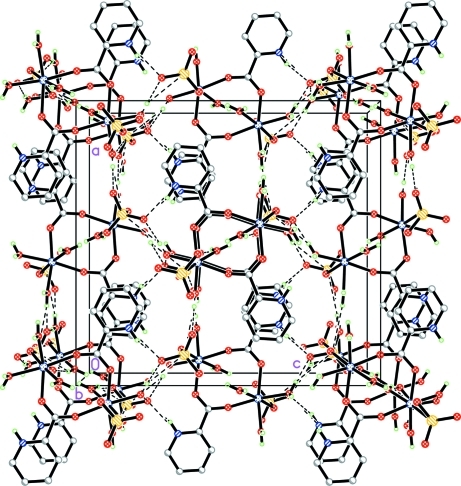
The packing diagram of the title compound viewed down the b-axis showing three dimensional networks. The dashed lines show the intermolecular interactions.

## supplementary crystallographic information

### Comment

As a part of a synthetic work on synthesis and characterization and applications (Lee *et al.,* 2004; Mautner *et al.,* 1997), of new Mn(II) complexes with aromatic carboxylic acid, we determined the X-ray structure of the title compound.

The molecule structure of the title compound (Fig. 1) is composed of a centerosymmetric dimer of a Mn(II) complex with pyridine-2-carboxylic acid. The bond lengths (Allen *et al.,* 1987) and angles are within the normal ranges. The geometry around the Mn^II^ is a distorted octahedron which is coordinated by two oxygen atoms of the carboxylic ligand, an oxygen atom of the sulfate anion, and three oxygen atoms of the coordinated water molecules. Intermolecular N—H···O and O—H···O hydrogen bonds (Table 1), link neighboring molecules, forming three dimensional network (Fig. 2). The crystal structure is further stabilized by the intermolecular π-π interaction [Cg1···Cg1^i^ = 3.842 (2) Å, (i) 3/2 - *x*, -1/2 + *y*, *z*; Cg1 is the centroid of the (N1/C1–C5) ring.

### Experimental

The title compound was synthesized by adding 10 ml solution of pyridine-2-carboxylic acid (1.456 g, 11.832 mmol) to a 10 ml solution of manganese sulfate (2g, 11.832 mmol). The mixture was stirred for 24 h. Light-brown single crystals of the title compound suitable for *X*-ray structure determination were obtained by slow evaporation of the solvents at room temperature after 4 days.

### Refinement

All hydrogen atoms of C were positioned geometrically with C—H = 0.93 Å and included in a riding model approximation with U_iso_ (H) = 1.2 U_eq_(C). The N-bound H atom was located in a difference Fourier map and constrained to refine with the parent atom by U_iso_ (H) = 1.2 U_eq_(N). The hydrogen atoms of the water molecules were located in a difference Fourier map and constrained to refine with the parent atoms by U_iso_ (H) = 1.5 U_eq_(O).

### Crystal data

**Table:**

[Mn_2_(SO_4_)_2_(C_6_H_5_NO_2_)_2_(H_2_O)_6_]	*F*(000) = 1336
*M**_r_* = 656.32	*D*_x_ = 1.879 Mg m^−^^3^
Orthorhombic, *P**b**c**a*	Mo *K*α radiation, λ = 0.71073 Å
Hall symbol: -P 2ac 2ab	Cell parameters from 2525 reflections
*a* = 16.886 (3) Å	θ = 2.5–27.4°
*b* = 7.6022 (15) Å	µ = 1.36 mm^−^^1^
*c* = 18.070 (4) Å	*T* = 291 K
*V* = 2319.6 (8) Å^3^	Block, light-brown
*Z* = 4	0.28 × 0.22 × 0.18 mm

### Data collection

**Table:**

Bruker SMART APEXII CCD area-detector diffractometer	1945 independent reflections
Radiation source: fine-focus sealed tube	1386 reflections with *I* > 2σ(*I*)
graphite	*R*_int_ = 0.033
φ and ω scans	θ_max_ = 25.0°, θ_min_ = 2.4°
Absorption correction: multi-scan (*SADABS*; Bruker, 2005)	*h* = −17→20
*T*_min_ = 0.702, *T*_max_ = 0.792	*k* = −8→9
5787 measured reflections	*l* = −21→18

### Refinement

**Table:**

Refinement on *F*^2^	Primary atom site location: structure-invariant direct methods
Least-squares matrix: full	Secondary atom site location: difference Fourier map
*R*[*F*^2^ > 2σ(*F*^2^)] = 0.028	Hydrogen site location: inferred from neighbouring sites
*wR*(*F*^2^) = 0.075	H atoms treated by a mixture of independent and constrained refinement
*S* = 1.26	*w* = 1/[σ^2^(*F*_o_^2^) + (0.0261*P*)^2^] where *P* = (*F*_o_^2^ + 2*F*_c_^2^)/3
1944 reflections	(Δ/σ)_max_ = 0.001
181 parameters	Δρ_max_ = 0.28 e Å^−^^3^
9 restraints	Δρ_min_ = −0.24 e Å^−^^3^

### Special details

**Table:**

Geometry. All esds (except the esd in the dihedral angle between two l.s. planes) are estimated using the full covariance matrix. The cell esds are taken into account individually in the estimation of esds in distances, angles and torsion angles; correlations between esds in cell parameters are only used when they are defined by crystal symmetry. An approximate (isotropic) treatment of cell esds is used for estimating esds involving l.s. planes.
Refinement. Refinement of F^2^ against ALL reflections. The weighted R-factor wR and goodness of fit S are based on F^2^, conventional R-factors R are based on F, with F set to zero for negative F^2^. The threshold expression of F^2^ > 2sigma(F^2^) is used only for calculating R-factors(gt) etc. and is not relevant to the choice of reflections for refinement. R-factors based on F^2^ are statistically about twice as large as those based on F, and R- factors based on ALL data will be even larger. the reflection "2 0 0" was removed because it was affected by the beam stop

### Fractional atomic coordinates and isotropic or equivalent isotropic displacement parameters (Å^2^)

**Table:**

	*x*	*y*	*z*	*U*_iso_*/*U*_eq_	
C1	0.80634 (14)	0.4943 (3)	0.39991 (16)	0.0274 (6)	
C2	0.73913 (18)	0.5011 (4)	0.44213 (18)	0.0441 (8)	
H2	0.7429	0.5010	0.4935	0.053*	
C3	0.66613 (19)	0.5082 (5)	0.4087 (2)	0.0608 (10)	
H3	0.6204	0.5132	0.4373	0.073*	
C4	0.66096 (19)	0.5077 (5)	0.3323 (2)	0.0580 (10)	
H4	0.6119	0.5134	0.3089	0.070*	
C5	0.72870 (19)	0.4990 (4)	0.29191 (19)	0.0513 (9)	
H5	0.7263	0.4971	0.2405	0.062*	
C6	0.88940 (16)	0.4876 (3)	0.43176 (16)	0.0281 (6)	
Mn1	1.06804 (2)	0.42796 (5)	0.39782 (2)	0.02431 (11)	
N1	0.79903 (13)	0.4929 (3)	0.32634 (13)	0.0341 (6)	
H1	0.8413	0.4879	0.2999	0.041*	
O1	0.94438 (10)	0.5098 (3)	0.38689 (11)	0.0369 (5)	
O2	1.10645 (13)	0.5419 (2)	0.50058 (11)	0.0407 (5)	
O3	1.11498 (11)	0.6541 (2)	0.34100 (11)	0.0326 (5)	
O4	1.03729 (12)	0.8763 (2)	0.40293 (11)	0.0413 (5)	
O5	1.08436 (11)	0.9258 (2)	0.28009 (10)	0.0364 (5)	
O6	1.17596 (11)	0.9245 (2)	0.38020 (12)	0.0439 (6)	
S1	1.10322 (4)	0.84382 (8)	0.35237 (4)	0.02353 (15)	
O1W	1.04261 (13)	0.2883 (3)	0.29403 (12)	0.0443 (6)	
H11	1.0473 (18)	0.183 (2)	0.2892 (18)	0.066*	
H12	1.0020 (14)	0.322 (3)	0.2717 (18)	0.066*	
O2W	1.02785 (15)	0.2033 (3)	0.46116 (13)	0.0499 (6)	
H21	1.023 (2)	0.104 (3)	0.4449 (17)	0.075*	
H22	1.007 (2)	0.208 (4)	0.5024 (12)	0.075*	
O3W	1.18013 (11)	0.2837 (3)	0.38886 (16)	0.0541 (7)	
H31	1.1775 (19)	0.177 (2)	0.385 (2)	0.081*	
H32	1.2266 (12)	0.317 (4)	0.386 (2)	0.081*	

### Atomic displacement parameters (Å^2^)

**Table:**

	*U*^11^	*U*^22^	*U*^33^	*U*^12^	*U*^13^	*U*^23^
C1	0.0269 (15)	0.0338 (14)	0.0214 (16)	0.0010 (10)	0.0006 (13)	−0.0029 (13)
C2	0.0358 (19)	0.069 (2)	0.0275 (18)	0.0012 (15)	0.0040 (15)	−0.0017 (15)
C3	0.0265 (17)	0.092 (3)	0.064 (3)	0.0010 (16)	0.0128 (19)	−0.004 (2)
C4	0.0307 (19)	0.084 (3)	0.060 (3)	0.0043 (16)	−0.0178 (18)	−0.008 (2)
C5	0.041 (2)	0.079 (2)	0.034 (2)	0.0091 (16)	−0.0136 (16)	−0.0051 (17)
C6	0.0309 (16)	0.0268 (15)	0.0268 (17)	−0.0002 (11)	−0.0045 (14)	−0.0049 (12)
Mn1	0.02487 (19)	0.0253 (2)	0.0228 (2)	−0.00078 (16)	0.0004 (2)	0.0016 (2)
N1	0.0271 (13)	0.0522 (15)	0.0231 (14)	0.0037 (10)	0.0008 (10)	−0.0015 (11)
O1	0.0254 (11)	0.0471 (12)	0.0381 (13)	0.0007 (8)	−0.0027 (10)	0.0033 (9)
O2	0.0474 (13)	0.0493 (14)	0.0254 (12)	−0.0007 (10)	−0.0098 (10)	−0.0025 (10)
O3	0.0453 (13)	0.0190 (10)	0.0336 (12)	−0.0011 (8)	0.0102 (9)	−0.0012 (9)
O4	0.0517 (12)	0.0352 (12)	0.0369 (12)	0.0031 (8)	0.0185 (11)	−0.0012 (10)
O5	0.0460 (12)	0.0352 (11)	0.0280 (11)	−0.0014 (9)	−0.0035 (9)	0.0077 (9)
O6	0.0364 (11)	0.0309 (11)	0.0643 (16)	0.0000 (9)	−0.0172 (10)	−0.0037 (11)
S1	0.0258 (3)	0.0226 (3)	0.0222 (4)	−0.0006 (3)	0.0009 (3)	0.0009 (3)
O1W	0.0555 (14)	0.0397 (12)	0.0376 (13)	0.0123 (10)	−0.0153 (11)	−0.0104 (11)
O2W	0.0808 (17)	0.0247 (12)	0.0441 (15)	−0.0037 (11)	0.0313 (13)	0.0021 (11)
O3W	0.0279 (10)	0.0296 (11)	0.105 (2)	0.0007 (8)	0.0048 (14)	−0.0058 (14)

### Geometric parameters (Å, °)

**Table:**

C1—N1	1.335 (3)	Mn1—O1	2.1878 (18)
C1—C2	1.368 (4)	Mn1—O3W	2.1934 (19)
C1—C6	1.517 (3)	Mn1—O1W	2.198 (2)
C2—C3	1.374 (4)	N1—H1	0.8600
C2—H2	0.9300	O2—C6^i^	1.245 (3)
C3—C4	1.383 (5)	O3—S1	1.4703 (17)
C3—H3	0.9300	O4—S1	1.4613 (19)
C4—C5	1.358 (5)	O5—S1	1.4817 (19)
C4—H4	0.9300	O6—S1	1.4622 (19)
C5—N1	1.342 (4)	O1W—H11	0.811 (17)
C5—H5	0.9300	O1W—H12	0.835 (17)
C6—O1	1.244 (3)	O2W—H21	0.817 (17)
C6—O2^i^	1.245 (3)	O2W—H22	0.827 (17)
Mn1—O2	2.1492 (19)	O3W—H31	0.818 (17)
Mn1—O3	2.1536 (18)	O3W—H32	0.826 (17)
Mn1—O2W	2.1652 (19)		
			
N1—C1—C2	118.6 (3)	O1—Mn1—O3W	163.57 (8)
N1—C1—C6	117.6 (2)	O2—Mn1—O1W	172.34 (8)
C2—C1—C6	123.8 (3)	O3—Mn1—O1W	92.91 (8)
C1—C2—C3	120.0 (3)	O2W—Mn1—O1W	90.50 (9)
C1—C2—H2	120.0	O1—Mn1—O1W	82.76 (8)
C3—C2—H2	120.0	O3W—Mn1—O1W	82.19 (9)
C2—C3—C4	119.7 (3)	C1—N1—C5	122.9 (3)
C2—C3—H3	120.1	C1—N1—H1	118.5
C4—C3—H3	120.1	C5—N1—H1	118.5
C5—C4—C3	118.9 (3)	C6—O1—Mn1	127.94 (18)
C5—C4—H4	120.6	C6^i^—O2—Mn1	142.42 (18)
C3—C4—H4	120.6	S1—O3—Mn1	131.82 (11)
N1—C5—C4	119.9 (3)	O4—S1—O6	110.72 (12)
N1—C5—H5	120.1	O4—S1—O3	110.86 (10)
C4—C5—H5	120.1	O6—S1—O3	110.26 (11)
O1—C6—O2^i^	128.5 (3)	O4—S1—O5	108.44 (12)
O1—C6—C1	116.0 (2)	O6—S1—O5	107.90 (12)
O2^i^—C6—C1	115.5 (3)	O3—S1—O5	108.57 (11)
O2—Mn1—O3	88.79 (7)	Mn1—O1W—H11	123 (2)
O2—Mn1—O2W	87.48 (9)	Mn1—O1W—H12	115 (2)
O3—Mn1—O2W	175.65 (9)	H11—O1W—H12	109 (2)
O2—Mn1—O1	104.56 (8)	Mn1—O2W—H21	125 (2)
O3—Mn1—O1	94.65 (7)	Mn1—O2W—H22	125 (2)
O2W—Mn1—O1	88.46 (9)	H21—O2W—H22	109 (2)
O2—Mn1—O3W	90.28 (9)	Mn1—O3W—H31	117 (2)
O3—Mn1—O3W	92.65 (8)	Mn1—O3W—H32	132 (2)
O2W—Mn1—O3W	85.13 (9)	H31—O3W—H32	111 (3)
			
N1—C1—C2—C3	−0.7 (4)	O3—Mn1—O1—C6	−145.0 (2)
C6—C1—C2—C3	179.5 (3)	O2W—Mn1—O1—C6	32.0 (2)
C1—C2—C3—C4	0.2 (5)	O3W—Mn1—O1—C6	98.9 (4)
C2—C3—C4—C5	0.5 (5)	O1W—Mn1—O1—C6	122.7 (2)
C3—C4—C5—N1	−0.8 (5)	O3—Mn1—O2—C6^i^	−176.0 (3)
N1—C1—C6—O1	11.2 (3)	O2W—Mn1—O2—C6^i^	1.7 (3)
C2—C1—C6—O1	−169.0 (3)	O1—Mn1—O2—C6^i^	89.5 (3)
N1—C1—C6—O2^i^	−167.9 (2)	O3W—Mn1—O2—C6^i^	−83.4 (3)
C2—C1—C6—O2^i^	11.9 (4)	O2—Mn1—O3—S1	−51.74 (16)
C2—C1—N1—C5	0.4 (4)	O1—Mn1—O3—S1	52.77 (16)
C6—C1—N1—C5	−179.8 (2)	O3W—Mn1—O3—S1	−141.97 (17)
C4—C5—N1—C1	0.3 (5)	O1W—Mn1—O3—S1	135.73 (16)
O2^i^—C6—O1—Mn1	21.0 (4)	Mn1—O3—S1—O4	−12.1 (2)
C1—C6—O1—Mn1	−157.90 (17)	Mn1—O3—S1—O6	110.89 (16)
O2—Mn1—O1—C6	−55.0 (2)	Mn1—O3—S1—O5	−131.11 (14)

Symmetry codes: (i) −*x*+2, −*y*+1, −*z*+1.

### Hydrogen-bond geometry (Å, °)

**Table:**

*D*—H···*A*	*D*—H	H···*A*	*D*···*A*	*D*—H···*A*
N1—H1···O5^ii^	0.86	1.97	2.799 (3)	161.
O1W—H11···O5^iii^	0.81 (2)	2.06 (2)	2.856 (3)	168 (3)
O1W—H12···O5^ii^	0.84 (2)	1.91 (2)	2.735 (3)	173 (3)
O2W—H21···O4^iii^	0.82 (2)	1.90 (2)	2.704 (3)	167 (4)
O2W—H22···O4^i^	0.83 (2)	1.97 (2)	2.758 (3)	158 (3)
O3W—H31···O6^iii^	0.82 (2)	1.92 (2)	2.736 (3)	177 (4)
O3W—H32···O6^iv^	0.83 (2)	1.84 (2)	2.660 (3)	172 (3)

Symmetry codes: (ii) −*x*+2, *y*−1/2, −*z*+1/2; (iii) *x*, *y*−1, *z*; (i) −*x*+2, −*y*+1, −*z*+1; (iv) −*x*+5/2, *y*−1/2, *z*.
